# Error in Main Text, Figures, Tables, and Supplement

**DOI:** 10.1001/jamanetworkopen.2025.23111

**Published:** 2025-06-24

In the Original Investigation titled “Estimated Vaccine Effectiveness for Respiratory Syncytial Virus–Related Lower Respiratory Tract Disease,”^1^ published December 13, 2024, there were errors in the main text, Figures, Tables, and Supplement. Cases of acute respiratory illness were erroneously categorized as lower respiratory tract disease. After reanalysis, individual data points were adjusted, but overall findings did not change. The authors have explained what happened in a Comment.^2^ This article has been corrected.^1^
